# The Role of Family Function and Triadic Interaction on Preterm Child Development—A Systematic Review

**DOI:** 10.3390/children9111695

**Published:** 2022-11-05

**Authors:** Johanna Larsson, Lisa Nyborg, Elia Psouni

**Affiliations:** Department of Psychology, Lund University, 221 00 Lund, Sweden

**Keywords:** preterm, family interaction, family functioning, family alliance, triadic interaction, child development

## Abstract

Preterm infants are at high risk of developmental disability/delay and are more dependent on their caregiving environment for regulation due to their neurological immaturity. A premature birth is also a major stressor to the family system that constitutes the infant’s caregiving environment. The following systematic review investigates whether families with preterm children differ from families with full-term children in their interactions, and what impact the quality of family interaction has on child development. Using the Cochrane model, we conducted a systematic review of quantitative studies published in psycINFO, socINDEX, and PubMed, concerning family quality in triadic interactions in families with premature infants and children, and at least one child development outcome variable. The quality of these studies was assessed using the Newcastle–Ottawa scale assessment form for cohort studies (NOS). Eleven studies were included in the review. Quality of family interactions is either equal to or poorer in families with preterm children, compared with families with full-term children. Importantly, the link between quality of family interactions and child development outcome is stronger in preterm children compared with full-term children, regarding both positive and negative influence. Our results highlight the importance of strengthening family interactions in order to promote development in preterm children. Notably, this review provides the first systematic overview of family function and the quality of triadic interactions in preterm families. The limited number of studies with a family-system focus makes it difficult for us to draw any definitive conclusions, while underscoring the need for more observational studies, particularly post-infancy, to be able to identify specific aspects of family interactions that may be critical for preterm child development.

## 1. Introduction

Approximately one in ten children are born prematurely. Premature birth is associated with an increased risk of physical illness, psychopathology, and neuropsychiatric developmental disorders [1]. Historically, prematurity has thus been construed mainly as an individual risk factor which renders the child more susceptible to negative influences in his/her environment. However, research suggests that prematurity can manifest itself as an increased sensitivity to both negative and positive environmental influences [2,3,4,5,6], supporting the so-called ‘differential susceptibility model’ [7]. Given their neurological immaturity, premature infants are more dependent on their caregiving environment for regulation, and thus more sensitive to the quality of this regulation.

Much of the research attesting the risks associated with prematurity has focused on a limited part of the child’s developmental environment; mainly the mother–child interaction [8,9,10,11], and more recently the father–child interaction [12,13,14]. However, in order to understand the long-term effects of prematurity, it is important to consider the multiplicity of family interactions within the caregiving environment. Inspired by the family systems theory [15], some studies in recent years have therefore focused on triadic interactions and family systems [16,17,18]. In the family systems theory, family members are seen as interdependent parts of a larger system, wherein each individual member serves as a co-creator in the processes and patterns that regulate family behavior. Therefore, studying individuals or dyadic interactions—such as the mother–child interaction—outside of their context, may give rise to fragmented and misleading information [15]. Notably, the triadic relationship (for instance, mother–father–child interactions) can support and strengthen dyadic interaction (for instance the mother–child interaction), as a third party can reduce stress and tension in the dyad [19]. Indeed, parents behave more responsibly in triadic interactions, and strong family alliances are associated with more responsive parenting [20].

Research suggests that the quality of family interactions has a considerable influence on a range of emotional, social, behavioral, and relational outcomes in the development of full-term children. Higher-quality family interactions predict better outcomes on perspective-taking ability, emotional regulation, emotional understanding, and social ability [21,22], while low levels of harmony within the family predict aggressive child behavior later on, after controlling individual factors and marital functioning [23].

The following systematic review will attempt to identify and examine research material with a systemic understanding and operationalization of the premature infant’s developmental environment. The purpose is to describe the quality of family interactions in the presence of a preterm-born child, and the potential significance these interactions have in the premature infant’s developmental abilities.

## 2. Material and Methods

### 2.1. Search Strategy

The search strategy was in accordance with the Cochrane model for systematic reviews [24]. Study protocol followed the preferred reporting items for systematic reviews and meta-analyses (PRISMA) [25]. The protocol was, however, not preregistered. Several pilot searches were conducted in the utilized databases (psycINFO, socINDEX, and PubMed), including both free text and controlled keywords. Relevant index terms were organized into search blocks for population, situation, and outcome, respectively. Index terms within search blocks were combined with “OR” and search blocks were combined with “AND.” We conducted the main search separately for each study question. The first search combined the search blocks “population” AND “situation” while the second search combined all three blocks (Table 1). We also conducted manual searches in reference lists and bibliographies.

Inclusion criteria were formulated prior to the selection process [24], with regard to study population, focus, and measurement of child outcome. Studies were included if they:

1. Concerned (a) premature infants (gestational age < 37 weeks) and/or infants with low birth weight (<2500 g) and (b) a comparison group with full-term children.
2. Quantitatively analyzed family interactions (>2 individuals), with measurements made during childhood (up to 11 years), based on: (a) validated direct observation or video recording; and/or (b) self-assessment measures with good psychometric properties; and/or (c) structured interviews that after coding provided quantitative outcome measures.
3. Reported a developmental outcome measure for the child based on parent and teacher assessments, self-assessments, observation, or psychometric testing.

Studies that either reported measures of dyadic interactions alone, exclusively operationalized the family environment in material or demographic terms, or did not report a distinct measure of family interaction were excluded. If 2 studies were based on the same sample and reported the same outcome measure, 1 of them was excluded. Literature reviews and studies based on qualitative methods or intervention designs were excluded. We also excluded duplicates, book chapters, articles that were not peer-reviewed, and articles published in languages other than Swedish or English.

### 2.2. Approaches to Studying the Family System

In the present work, we use the term “quality of family interaction” to denote a relational measure at group level, hoping to capture the family members’ way of functioning together. Family processes may be conceptually divided into actual behaviors and interaction patterns (the ‘practicing family’), and the family members’ perceptions and thoughts about behaviors and interactions in the family (the ‘represented family’) [26]. Notably, the earliest and most common methodologies in this field of research address the represented family, through self-assessments typically answered by the mother (like the first 4 instruments in the selected studies, below) and interviews that measure a family member’s perception of family function [27,28,29,30,31,32,33,34]. However, a more recently developed branch of methodology measures the practicing family, through observation and coding of interactions [35,36,37] (see the final 3 instruments below).

### 2.3. Instruments in Selected Studies

#### 2.3.1. Family Environment Scale (FES)

The scale comprises 90 items organized in 10 subscales divided into 3 domains. The first domain covers relationship scales; cohesion (α = 0.78), expressiveness (α = 0.69), and conflict (α = 0.75). The second domain covers scales measuring personal development goals; independence (α = 0.61), performance orientation (α = 0.61), intellectual-cultural orientation (α = 0.78), recreation/recovery (α = 0.67), and religious/moral attitude (α = 0.78). The third domain covers family structure scales; organization (α = 0.76) and control (α = 0.67). Subscale scores combine into a profile of family environment [38].

#### 2.3.2. Family Relation Scale (FARS)

FARS was adapted for use in Sweden [39] and took inspiration from the American family adaptability and cohesion evaluation scale (FACES) [40]. FARS consists of 46 statements which correspond to a five-point scale (almost always–almost never), which probes family function in terms of attribution, joint activity, isolation, chaos, and over-involvement, with high internal consistency α = 0.9016.

#### 2.3.3. Nordic Health and Family Questionnaire (NHFQ)

The questionnaire contains several subscales including parent education, family income, and family structure. One subscale (12 items) measures family functioning with respect to problem solving, effective responsiveness, effective involvement, communication and roles, and behavioral control. Responses are on a 5-point scale (totally disagree–totally agree). NHFQ was validated in a dissertation [31].

#### 2.3.4. Family Assessment Device (FAD)

The scale comprises 60 items that measure 7 dimensions of family functioning based on the McMaster model of family functioning [41], including problem solving, communication, roles, effective responsiveness, effective involvement, behavior control, and a dimension for general functioning. Internal consistency ranged from α = 0.71 to α = 0.92 [42], with some variation between subscales [43].

#### 2.3.5. Coding Interactive Behavior Manual (CIB)

CIB [44] codes interaction between parents and children based on 42 items scored on a 5-point scale. CIB has been validated in studies of both well-functioning dyads and dyads in risk groups and has a high degree of sensitivity to infant age, biological and socio-emotional risk, psychopathology in the mother, and intervention effect [45,46,47]. Feldman [35] used CIB to code triadic interactions during semi-structured play with an inter-rater reliability of 93%.

#### 2.3.6. Family Alliance Assessment Scale (FAAS)

FAAS is an instrument for coding observations of family interactions, through 11 scales mainly focused on the non-verbal behavior of family members. The scales capture posture and gaze, inclusion of partners, casting, joint structuring, parental support, warmth in the family, validation of the child’s experience, authenticity in effects, interactive mistakes and solutions during activity, and interactive mistakes and solutions during transitions. Internal consistency is α = 0.92 [21]. Two studies [36,37] used FAAS for coding LTP with an inter-rater reliability of 83–93%.

#### 2.3.7. Home Atmosphere

Home atmosphere measures the emotional climate in the family and includes 7 items concerning acceptance, demands, control, encouragement, and communication, based on items and categories from various other scales and interview guides, including FES. Measured aspects receive a score of 0 (unfavorable), 1 (tolerable), or 2 (advantageous). Reported inter-rater reliability is between 84% and 96% [34].

### 2.4. Quality Assessment

We assessed the quality of the articles using the Newcastle–Ottawa scale assessment form for cohort studies (NOS) [48]. The NOS form assesses the quality of non-randomized studies through a rating system that allows the assessor to define relevant factors to control for in the given context, as well as the appropriate length for follow-up. This was deemed relevant for the present review given the fact that development in children takes place in continuous interaction with the environment. The scale consists of 8 criteria divided into 3 domains: selection, comparability, and outcome. In total, an article can be given 9 stars, with 6 stars (3 stars for selection, 1 for comparability, and 2 for outcomes) indicating good quality. In accordance with the Cochrane model [24], 2 reviewers independently assessed the quality of each paper. Consensus was reached through discussion and consultation with a third party.

For the domain selection, studies receive credit (a star) for (1) representativeness of the exposed cohort, (2) selection of the non-exposed cohort, (3) ascertainment of exposure, and (4) demonstration that the outcome of interest was not present at baseline. The last criterion is not applicable to the studies in this literature review and was thus not considered. For comparability, credit is given for (5) comparability of cohorts on the basis of design or analysis. Studies were given 1 star if they controlled for basic characteristics such as participants’ age and gender, and another star if they controlled for additional relevant factors (family socio-economic status, parents’ level of education and mental health, family structure, child’s medical risk). For the domain outcomes, studies can receive 3 stars, for (6) assessment of outcomes, (7) follow-up long enough for the outcomes to occur, and (8) adequate follow-up of the cohorts. Follow-ups of approximately 6 months or longer were deemed adequate.

### 2.5. Data Collection

Two reviewers collected data from the included articles into standardized forms [14], summarizing background information on the year of study, nationality, participant characteristics, method, and instruments (Table S1) and information on outcome variables and study results.

### 2.6. Data Analysis

Given the fact that family interactions are likely to adjust developmentally to children’s changing needs, we analyzed results separately for different age groups. We also made a methodological division of the studies, maintaining the conceptual distinction between represented and practicing family. To answer the first research question, results concerning family functioning or quality in triadic interactions in families with premature infants were compared with results from families with full-term children, first overall and then separately, for different age groups and methodologies. To answer the second research question, we focused on the link between family functioning or quality in triadic interactions and developmental outcome measures, first comparing premature and full-term infants, and then considering age and method.

## 3. Results

### 3.1. Selection of Studies

**Figure 1:**
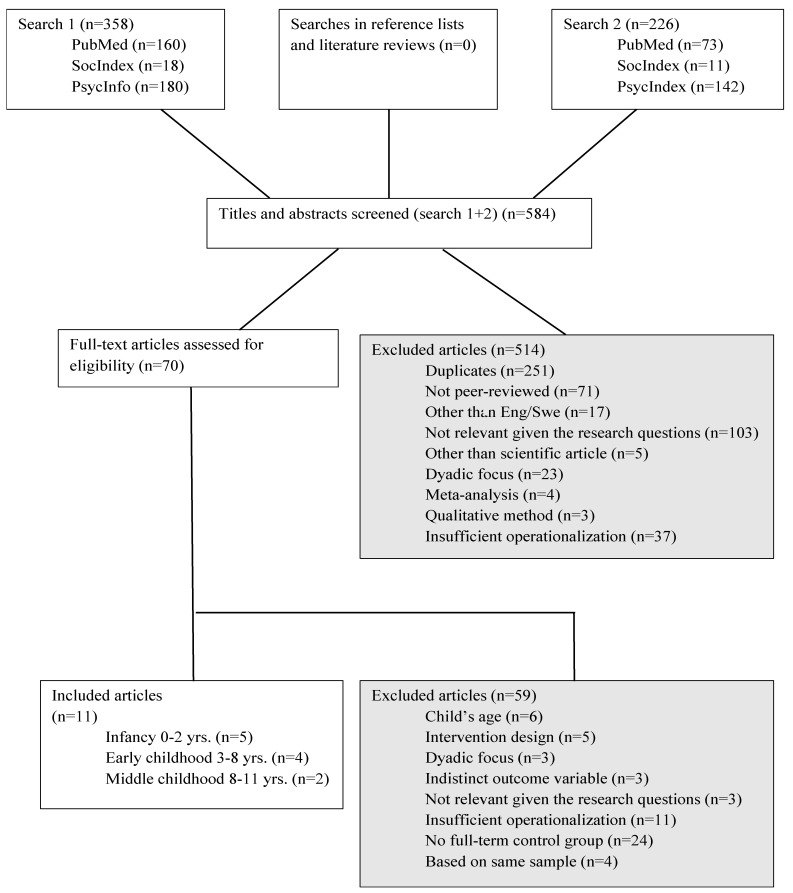
Summary of search procedures.

The searches resulted in 584 articles. In accordance with the Cochrane model [24], and as a first step of assessing suitability for inclusion, two authors individually screened the title and abstract of each article. We then compared our assessments. If disagreement arose, the third author was consulted. Of the 584 articles, 514 were excluded after title and abstract screening. For the next step, 70 articles were screened in full text, and 59 were excluded (see Figure 1: Excluded articles), resulting in a final sample of 11 studies.

### 3.2. Description of Included Studies

The included studies were published between 1987–2018 (Table 2) and were undertaken in three different countries in Europe, as well as the USA, Australia, and Israel. Of the 11 studies, four concerned families with children in the infant period (0–2 years), five concerned children in early childhood (3–8 years), and two concerned children in late childhood (8–11 years). Eight studies included all degrees of prematurity, from moderate to extreme, while three studies focused on extremely preterm infants. Most studies included all levels of low birth weight (low to extremely low), while one study included only children with extremely low birth weight. In all studies, the control group consisted of full-term children with normal birth weight (>2500 g).

To investigate family interactions, seven studies used self-assessment instruments, one study conducted a semi-structured interview, and three studies used observation. All three observational studies examined families with infants (0–2 years), while the studies using self-assessment examined families with children from infancy to late childhood (0–11 years). The self-assessment instruments used were FAD (n = 3), FARS (n = 2), FES (n = 2), and NHFQ (n = 1). In seven out of eight studies, the self-assessment form was answered by the families’ mothers. Only one study included responses from both parents and found no differences in how mothers and fathers assess family functioning [30]. Of the three observational studies, two were based on semi-structured play coded with FAAS, while the third was based on unstructured play coded with CIB. Four studies examined children’s cognitive development, three studies examined social and linguistic development, and one study examined the prevalence of psychopathology. There was great variation in the instruments used for measuring child development. Only Bayley-III appeared in several studies (n = 3).

The quality of the included studies was essentially good (Table 3). Two studies were assessed to be of poor quality, in one study this was due to a high dropout rate during follow-up (compromising outcome), and in another study the comparison group comprised of older children than the original study group (compromising comparability).

### 3.3. Quality of Family Interaction

The first purpose of the systematic review was to examine family interactions in the context of prematurity. Of 11 included studies, five studies showed significant group differences regarding family functioning [29,32,34,35,37] (Table S1). Four studies showed poorer family functioning in families with premature children, while results from Kalmár [34] suggested higher family functioning (home atmosphere scores) in families with premature children. Five studies showed no group difference regarding family functioning [27,28,30,31,36], and one study reported no results for group differences [33].

#### 3.3.1. Children 0–2 Years

Two out of three observational studies showed poorer quality of triadic interactions in families with premature infants compared with families with full-term infants [35,37]. Feldman [35] reported lower levels of cohesion and higher rigidity. One study [37] reported a poorer overall quality in triadic interactions, but no difference in co-parenting quality and parental alliance. The third observational study showed no differences between groups in quality of triadic interactions [36]. One self-assessment study [27] found differences in how family functioning is linked to other variables. In families with premature infants, family functioning was strongly linked to mother–infant behavior, particularly concerning affective interaction between mother and child [27]. However, this was not the case in families with full-term infants. The second study using self-assessment reported no data with relevance to quality of family interaction [33].

#### 3.3.2. Children 3–8 Years

Three studies showed group differences regarding family functioning, but with inconsistent results [29,32,34]. The fourth study showed no differences [30]. One study [32] showed that families with premature children reported poorer overall family functioning when compared with families with full-term children, including problem-solving difficulties, less direct communication, less established, unclear, and unequal roles, limited effective responses, and more involvement in each other’s actions. The pattern was consistent over time: low scores for family functioning at two years of age predicted low scores for family functioning at seven years of age in both groups (*p* = 0.13) [32]. One study [29] found that families with premature children reported general difficulties, one in particular being the inability to perceive others as separate individuals and for the family members not to constantly intrude in each other’s space. Notably, the study compared families with premature children aged three, five, and eight with a norm group consisting of children aged 9–15. The third study reported that families with premature children evaluated their home atmosphere more positively (higher scores) than the control families [34]. Finally, one study [30] showed no significant group differences in family functioning.

#### 3.3.3. Children 9–11 Years

None of the included studies found any group differences in functioning in families with older children [28,31].

### 3.4. Quality of Family Interaction and Child Development

The second purpose was to compare the role that quality of family interactions plays in the development of premature and full-term children, respectively. Of 11 studies included, five examined the relationship between family functioning and some aspect of child development, see Table S1. The aspects examined were cognitive, social, and language development. One study also examined the relationship between family functioning and psychopathology [31]. Three of these studies found a relationship between family functioning and outcome on developmental measures, but only in families of premature children [27,31,36]. One study found a positive link between family functioning and IQ among both premature and full-term infants [34]. One study found no links between family functioning and cognitive and social development, for premature or full-term children [33].

#### 3.4.1. Children 0–2 Years

Three studies examined family functioning and child development in children aged 0–2 years [27,33,36]. Two of these studies found a difference between premature and full-term infants. One [36] found that the quality of triadic interactions moderated the group difference in social functioning. Premature infants exposed to low-quality triadic interactions showed poorer social functioning compared to full-term infants, yet when exposed to high-quality triadic interactions, their social functioning was significantly better than that of full-term children. Regarding cognitive function, there was a link to quality in triadic interactions for all children. One study [27] reported a strong correlation between the level of organization within the family system and cognitive and language development at 12 months of age. At 24 months the connection remained significant for language, but not for overall cognitive development. These links were not found for full-term children. No link was found between family functioning and cognitive or language development [33].

#### 3.4.2. Children 3–8 Years

The study that examined family functioning and child development in children aged 3–8 years [34] found a positive relationship between child IQ, emotional climate, and social adjustment in the family, for premature children and full-term children alike. For full-term children, the link was significant for the entire age range. For premature children the link was significant only in the older ages.

#### 3.4.3. Children 9–11 Years

Farooqi et al. [31] examined family functioning and psychopathology in children aged 9–11 years old, finding a link only for premature children. Poorer family functioning was associated with higher levels of anxiety, somatic problems, and attentional problems.

## 4. Discussion

This is the first systematic review to examine interactions in the family system in the context of a premature birth, and the first one to relate the development of the premature child to the functioning of a family system. The review makes use of a systemic perspective and thus emphasizes the transactional aspect of the relationship between parents and children within the family system—the dialectical interactions between the child and its environment, positing bidirectional influences between nature and nurture that continuously modify each other [49].

### 4.1. Quality of Family Interaction in the Context of a Child Born Prematurely

Regarding quality of family interaction, we found support for both equivalent and poorer quality of interaction in families with premature children compared with families with full-term children.

The premature infant is born into a crisis. Being part of a premature birth is often a turbulent experience for the parents, as the material and psychological preparations for childbirth, as well as the pregnancy itself, end unexpectedly. Furthermore, the birth often involves increased medical care for the child and child–parent separation during the neonatal period. Consistently, symptoms of post-traumatic stress are common in parents of premature infants [50], and mothers of prematurely born infants are at a greater risk of developing postpartum depression compared with mothers of full-term infants [50,51]. Moreover, parents of premature infants experience higher levels of general and parental stress, which is linked to the immediate shock following the premature birth, prolonged hospital stay, and worries concerning current and future child behavior and development [50].

Thus, it is not surprising that the quality of family interactions is equivalent or poorer in families with premature infants compared with families with full-term children. Dyadic research has revealed similarities in parenting in terms of sensitivity and attachment [52], but also differences in interaction style [11,53,54]. Mothers of premature infants are more active and overstimulating when interacting with their infants [55], and a recent meta-analysis confirms that parents of premature infants who are more intrusive and controlling persist with this style of parenting throughout childhood [54]. Expanding the focus to triadic interactions in the family, one study in the present review found lower cohesion and more rigidity [35], while another found more enmeshment [29]. A rigid family style may contribute to an environment with limited freedom, task focus, intrusion, and competition.

As differences in interaction patterns cannot be explained by differences in maternal sensitivity, they may be caused by the characteristics and needs of the premature infant. Compared to mothers of full-term infants, mothers of premature infants attempt to direct their infants’ attention more often [53]. This high activation may be a compensation for the premature infant’s weak signals [56], difficulty to sustain attention and eye contact, and lower alertness [57]. Given that premature infants also smile less [58], have poorer regulation, a more challenging temperament [59], and often express negative effects such as anger or frustration [60], it may not be appropriate to mirror these expressions too much, as it risks contributing to more negative effects [61]. Thus, the differences in certain aspects of the parent–child interaction may be favorable, stemming from an adaptive process that is beneficial to the premature infant. For example, stricter family organization and control correlated positively and strongly with desirable developmental outcomes in the premature child [27].

Finally, an integrative meta-analysis found that premature children demonstrated secure attachments on equal terms with full-term children in the strange situation at 12 months of corrected age [11]. Compared to full-term infants, the premature infants’ first months appeared to be critical for their mothers’ interactive style, but these differences decreased after a few months, leaving no trace in the premature infants’ attachment at group level [11].

### 4.2. Quality of Family Interaction and Child Development

Regarding the significance of family interactions for the development of premature children, three out of five studies examining this found stronger links for premature children compared with full-term children [27,31,36]. Thus, the quality of family interactions appears more important for premature children than for full-term children, suggesting increased sensitivity of the premature infant in line with the differential susceptibility model [7]. The variation in the results does not appear to reflect systematic differences regarding child age, methodology, or measure used. However, further statistical analysis is required in order to draw definitive conclusions.

Results are different for other domains of family functioning. Synchronizing emotional experiences and sharing positive emotions in the parent–child interaction helps the child to develop social skills and, later on, the ability to self-regulate [18,35,47,62]. Although no differences have been shown between mothers of premature and full-term children in their ability to tune in and respond to the child’s signals [52,55], maternal sensitivity at 18 months predicted the child’s socio-emotional skills at 11 years for premature, but not full-term children. Premature infants benefit the most from effect sharing within the family, validation of the child’s emotional experiences, and authenticity in expressed effects [36]. Premature children showed better social ability in families with high-quality interactions, and larger handicap in families with low-quality interactions, when compared with the full-term group in the same type of triadic interactions [36], supporting the differential susceptibility model [7]. More studies that examine specific aspects of family interactions and their potential links to premature infant development are required.

### 4.3. Represented and Practicing Family

There is no consensus, and thus an abundance of overlapping constructs and operational definitions of quality, of family interaction in the field of systemic research. Lack of parsimony thus complicates comparisons and impedes further development in the field. Furthermore, triads or larger units are particularly difficult to observe systematically, which means they are often broken down into manageable dyads. However, triadic interactions are more complex than the sum of their dyadic interactions [15]. One observation method which registers and analyzes the triadic interactions between mother, father, and child is the Lausanne trilogue play (LTP) [63]. The method has inspired several studies, but is limited to three interaction parties, excluding siblings or other family members from the analysis. The semi-structured observation PicNic Game (PNG) [16] has the capacity to follow more than three interacting parts but lacks application in research.

As only three studies used an observational method [35,36,37] and one used a semi-structured interview [34], compared to seven studies based on self-assessment reports by mothers [27,28,29,30,31,32,33], the results of this review primarily concern the represented family, and in particular the mothers’ representations. We found no difference regarding quality of family interactions depending on whether the study examined the practicing or the represented family, but since observational studies are much less common than self-assessment studies, this result is not conclusive.

In self-report, respondents can only share information consciously available to them. From a systemic perspective, it would be desirable to consider more than one family members’ representation of the family. A study comparing mothers’ and fathers’ assessment of family functioning in families of premature infants found no significant difference between the parents’ assessments [64]. Other studies in non-clinical populations report that fathers tend to rate family functioning lower than mothers [65,66]. Differences in assessment of family functioning among members of the same family can itself be an indicator of poor family functioning [67]. Future systemic research on families with premature children should therefore include more family members’ representations of the family and to a greater extent focus on the practicing family.

### 4.4. Direction of Causality

A difficult but interesting question is the causal relationship between prematurity and family interaction. Is poor quality in family interactions itself a risk factor for prematurity? If so, how do these patterns of interaction develop after the premature infant is born? Poor family functioning implies an inflexible family system that has difficulty adapting to change. How does a premature birth affect a family system that already functions poorly? We know that different types of stress increase the risk of premature birth [68,69,70], and that over-involvement within the family captures variance in birth weight [71].

The question of causality is also relevant when considering child development. Can low quality family interactions be attributed to the premature infant’s developmental difficulties, or vice versa? Due to design limitations in the existing studies, the current review can only comment on causal relationships to a limited extent. However, research using intervention design has provided some evidence to suggest that the quality of triadic interactions affects child development. For instance, an intervention study aimed at strengthening the triadic interactions in families with premature children showed that improved interactions result in a significant reduction in parental stress and greater motor, language, and socio-emotional developmental outcomes in the intervention group [3]. Another intervention, intended to strengthen the triadic interaction with the help of video feedback, had positive results on full-term children’s communicative ability and motor development [72].

### 4.5. Limitations

The studies included in this systematic review differ in terms of child age and method, making comparisons difficult and posing limitations to generalizable conclusions. The review also included two studies assessed as being of poor quality. A larger number of studies of exclusively good quality would have provided a more robust basis for conclusions and clinical recommendations, but no such studies are yet available.

The included studies represent a breadth of constructs, methods, and instruments and reveal overlapping concepts and unclear distinctions, building on six different measurement methods and several different constructs, all intended to measure the quality of family interactions. While stricter inclusion criteria would have made a review of the subject impossible, the lack of parsimony in operationalization of quality of family interactions threatens construct validity. In the present paper, we made a conceptual distinction between the represented and the practiced family while assuming that these two methodological branches address overlapping phenomena. However, how the different instruments relate to each other is unclear.

The quality evaluation of the studies was based on the assessment method NOS, where assessors themselves determine important controls and adequate follow-up periods. Since the focus of the studies was the quality of family interactions, no baseline (pre-birth) measurements were available, which leads to removal of the respective criterion from the quality assessment. The necessary adaptation of the NOS arguably poses a risk of arbitrariness in the quality assessment, but it did not change the quality judgement for any of the studies.

NOS attributes low quality status to self-assessment questionnaires. However, a criterion that instead assesses the method’s suitability in relation to what is studied would have been preferable. For instance, when studying the family members’ subjective perceptions of their family, self-assessment is the most suitable method. Furthermore, instead of rejecting certain methods of measurement, the quality assessment could make demands on high levels of reliability and validity for these measurements. Importantly, domains for assessing the suitability for the statistical analyses given the research question, and ethical conduct, would have also been desirable. For instance, eight of the included studies named ethical approval, while three studies reported no such information.

The review did not include searches in the grey literature, which increases the risk of publishing bias. Finally, all included studies are based on samples from western, industrialized, and democratic countries, posing a risk of cultural bias. It is possible that the quality of family interactions is expressed differently in other cultural contexts and that operational definitions need to be adapted.

## 5. Conclusions

The current systematic review has gathered existing studies with a systemic perspective on premature families and provided an overview of comparisons of family interaction quality between families of premature and full-term children. Results indicate that the quality of family interactions is equal or poorer among families with premature children, compared with families with full-term children. Results further indicate that quality of family interactions is more crucial for premature than full-term children, concerning both gains and potential harms. This aligns with the differential susceptibility model and challenges the idea that premature infants are only burdened by risks. It also emphasizes the need for supportive and preventive intervention at the level of family interaction. To provide an evidence base for such intervention, future research ought to identify the aspects of family interactions that are uniquely crucial for different developmental outcomes.

## Figures and Tables

**Table 1:** Search terms used in each database.

Database	Population	Situation	Outcome
psycINFO	*Search terms:* prematurity OR “premature infant *” OR “born premature *” OR preterm OR “low birth weight” OR LBW OR VLBW OR ELBW OR “small for gestational age” *Index terms:* “premature birth” OR “birth weight”	*Search terms:* “family cohesion” OR “family cohesiv *” OR “family function” OR “family alliance” OR ”family enmeshment” OR ”coplaying” OR “family environment” OR “family interaction *” OR “Lausanne Trilogue Play” OR “triadic interaction *”	*Search terms:* “infant development *” OR “development * outcome *” OR “child development *” OR development *
socINDEX	*Search terms:* prematurity OR “premature infant *” OR preterm OR “very low birth weight” OR VLBW OR “extremely low birth weight” OR ELBW *Index terms:* “PREMATURE infants”	*Search terms:* “family function *” OR “family environment” OR “family interaction *”	*Search terms:* “infant development *” OR “development * outcome *” OR “child development *” OR development *
PubMed	*Search terms:* prematurity OR preterm OR “low birth weight” OR LBW OR VLBW OR ELBW OR “small for gestational age” *Index terms:* “Premature Birth” OR “Infant, Premature” OR “Infant, Extremely Premature” OR “Infant, Low Birth Weight”	*Search terms:* “family cohesion” OR “family cohesiv *” OR “family function *” OR “family alliance” OR “family enmeshment” OR “coplaying” OR “family environment” OR “family interaction *” OR “Lausanne Trilogue Play” OR “triadic interaction *” OR “mother–father–child” OR “triadic *” OR “family system”	*Search terms:* “infant development *” OR “child development *” OR development * Index terms: “child development”

Note. “*” after a search term denotes openness to combinations with any other terms.

**Table 2:** Included Articles.

Article	Country	Families (n)	Age	Degree of Prematurity	Birth Weight	Method	Measure	Construct	Development Measure
[27]	USA	70	1–24 months	p:26–36 w c:39–42 w	p < 1801 g k > 2500 g	Self-assessment	FES	Family environment	Bayley-III
[29]	Sweden	46	2–8 years	p: 23–27 w c: no information	No information	Self-assessment	FARS	Family function	
[31]	Sweden	172	11 years	p: M = 24.6 w, SD = 0.7 c: M = 39.2 w, SD = 2.7	p: M = 765 g, SD = 111 c: M = 3520 g, SD = 601	Self-assessment	NHFQ	Family function	CBCL TRF
[35]	Israel	145	4 months	p <33 w k >36 w	p < 1650 g k > 2500 g	Observation, unstructured play	Coding: CIB	Family cohesion Family rigidity	
[37]	Italy	78	19 months	p: M = 29.7 w, SD = 3.3 c:>37 w	p: M = 1294 g SD = 602 k > 2500 g	Observation, semi-structured play	LTP Coding: FAAS	Family alliance	
[36]	Israel	150	6–12 months	p: M = 32 w SD = 1.7 c: M = 39 w SD = 1.2	p: M= 1818 g SD = 475.8 c: M = 3322 g SD = 426.8	Observation, semi- structured play	LTP Coding: FAAS	Family alliance	ESCS WABS Bayley-III
[34]	Hungary	155	0–8 years	p: M = 33.6 w, SD = 2.2 k > 37 w	p: M= 1746 g, SD = 361 k > 2500 g	Semi- structured interview	Coding: Home atmosphere	Home atmosphere	Budapest-Bine test
[33]	USA	120	2–5 years	p: M = 26.6 w, SD = 1.8 c: M = 39.5 w, SD = 1.0	p: M = 943 g, SD = 258 c: M = 3372 g, SD = 484	Self-assessment	FAD	Family dysfunction	Bayley-III WPPSI-III CELF-P2 MABC-2
[30]	Sweden	78	4 years	p: M = 31 w, SD = 1 k > 37 w	p: M = 1740 g, SD = 190 k > 2500 g	Self-assessment	FARS	Family function	BSQ
[28]	USA	164	11 years	p: no information c: >37 w	<750 g (n = 60) 750 g–1499 g (n = 55) >2500 g (n = 49)	Self-assessment	FAD	Family function	
[32]	Australia	252	2–7 years	p: M = 27.5 w SD = 1.9 c: M = 39.1 w SD = 1.3	p: M = 969 g SD= 221 c: M = 3318 g SD = 510	Self-assessment	FAD	Family function	

**Table 3:** Quality assessment based on Newcastle Ottawa Scale.

Article	Selection (Max 3)	Comparability (Max 2)	Outcome (Max 3)	Total Quality
[27]	***	**	*	Poor
[29]	***		**	Poor
[31]	***	**	**	Good
[35]	***	**	***	Good
[37]	***	*	**	Good
[36]	***	**	***	Good
[34]	***	*	**	Good
[33]	***	*	**	Good
[30]	***	**	**	Good
[28]	***	**	**	Good
[32]	***	*	**	Good

Note: Stars-system is explained in detail in Section 2.4 Quality assessment.

## Data Availability

Not applicable.

## Supplementary Materials

The following supporting information can be downloaded at: https://www.mdpi.com/article/10.3390/children9111695/s1, Table S1: Summary of results in the studies [27,28,29,30,31,32,33,34,35,36,37].
