# Supplementary material for: The Role of Family Function and Triadic Interaction on Preterm Child Development—A Systematic Review
Source: Children (Basel). 2022 Nov 5;9(11):1695. doi: 10.3390/children9111695 (PMC9689109; doi:10.3390/children9111695)
Supplement: Supplementary file 1 [file children-09-01695-s001.zip › children-1987919-supplementary.pdf]

Table S1. Summary of results in the studies.

Article	Method	Difference FF P and K	FF and cognitive development (CD)	FF and social and language development (SLU)	FF and psychopathology (PP)
* Crnic & Greenberg, 1987	Self assessment (FES)	No difference in FF between P and C.	FF correlates with CD for P but not for C. 12 months. P(r=.39, p<.05) K(r=.22, is)	FF correlates with SLU for P but not for C. 12 months. P(r=.44, p<.05) C (r=-.03, ns) 24 months P(r=.38, p<.05) C(r=.11, ns)	x
Feldman, 2007b	Observation (CIB)	Yes. P<C Cohesion F=4.65 (p<.05) Rigidity F=19.09 (p<.01)	x	x	x
Gatta, 2017	Observation (FAAS)	Yes. P<C t=-5.971 (p<.01)	x	x	x
Gueron-Sela et al., 2015	Observation (FAAS)	No difference in FF between P and C.	FF correlates with CD for P but not for C. P(R=.35, p<.01) C(R=.17, is)	FF correlates with SLU for P but not for C. P(R=.42, p<.001) C(R=.01, ns)	x
Lean et al., 2018	Self assessment (FAD)	x	FF does not correlate with CD for P nor C. (p>.05)	FF does not correlate with SLU for P nor C. (p>.05)	x
Kalmár, 1996	Semi-stuctured interview (Home atmosphere)	Yes. P>C Home atmosphere F=4.1 (p<.05)	FF correlates with CD for P and for C. For P age 5-8 yrs, for C age 3-8 yrs. (p<.05)	x	x
Leijon, et al., 2003	Self assessment (FARS)	No difference in FF between P and C.	x	x	x
* Eriksson & Pehrsson, 2003	Self assessment (FARS)	Yes. P<C t=11.6 (p<.05)	x	x	x
Treyvaud et al., 2011	Self assessment (FAD)	Yes. P<C β=.14 (p=.04)	x	x	x
Farooqi et al., 2007	Self assessment (NHFQ)	No difference in FF between P and C.	x	x	FF has an effekt on PP for P but not for C. (p<.05).

Taylor et al., 2001	Self assessment (FAD)	No difference in FF between P and C.	x	x	x

Note.
FF = family functioning, P = premature group, C = control group, ns=non-significant, x=no presented outcome measurement, *=poor quality in NOS assessment
0-2 yrs 3-8 yrs 9-11 yrs
